# Effect of Packing Nonuniformity at the Fiber Bundle–Case Interface on Performance of Hollow Fiber Membrane Gas Separation Modules

**DOI:** 10.3390/membranes12111139

**Published:** 2022-11-13

**Authors:** Lili Sun, Grigorios Panagakos, Glenn Lipscomb

**Affiliations:** 1 National Energy Technology Laboratory, 626 Cochran Mill Road, Pittsburgh, PA 15236, USA; 2 NETL Support Contractor, 626 Cochran Mill Road, Pittsburgh, PA 15236, USA; 3 Department of Chemical and Environmental Engineering, University of Toledo, 2801 W. Bancroft Street, Toledo, OH 43606-3390, USA; 4 Department of Chemical Engineering, Carnegie Mellon University, 5000 Forbes Ave, Pittsburgh, PA 15213, USA

**Keywords:** hollow fiber membrane, membrane modules, post-combustion carbon capture, gas separations

## Abstract

High-fidelity simulations of momentum and mass transfer within a hollow fiber gas separation membrane module are here reported. The simulations capture the potential detrimental effects of poor fiber packing at the bundle–case interface on fluid distribution and performance. Results are presented for both circular and planar fiber bundles. The length over which bundle–case gaps affects flow is determined. The length increases dramatically with increasing fiber packing fraction. As the packing fraction approaches 0.6, the impact extends over the entire bundle diameter for small modules (<1000 fibers). The results clearly demonstrate the detrimental effect of poor packing along the case and can be used to develop module manufacturing guidelines. To reduce computational costs, an equivalent planar bundle module approximation is developed. The approximate simulations agree well with results from full 3-D simulations and can reduce computational costs without sacrificing fidelity.

## 1. Introduction

Carbon capture technology is of growing interest due to greenhouse gas emissions from fossil fuel-fired power plants, cement plants, and the petrochemical industry. The growth in CO_2_ emissions from the burning of fossil fuels has increased since 1960 [1]. While emissions dropped during the COVID pandemic, growth is expected to continue as the global economy reopens. Emission growth is predicted to lead to undesirable climate changes including temperature increases and extreme weather events [2]. Although a transition from fossil fuels to low-carbon energy is underway, a complete shift of the existing infrastructure to cleaner alternatives is not expected quickly. Thus, separation technologies that capture CO_2_ emissions at concentrated point sources or directly from air will play a vital role in bridging the gap until the new energy infrastructure is realized.

Membrane gas separation processes are cost-effective options for gas separations due to reduced energy consumption; the reduction in energy costs is partially offset by an increase in capital costs relative to competing technologies. Membrane processes offer other advantages too, including smaller footprints and rapid response times. Membrane technology for carbon dioxide capture from concentrated carbon dioxide sources, such as power and cement plants, is being developed and tested at the pilot scale [3,4] and is a promising option for capture.

**Figure 1:**
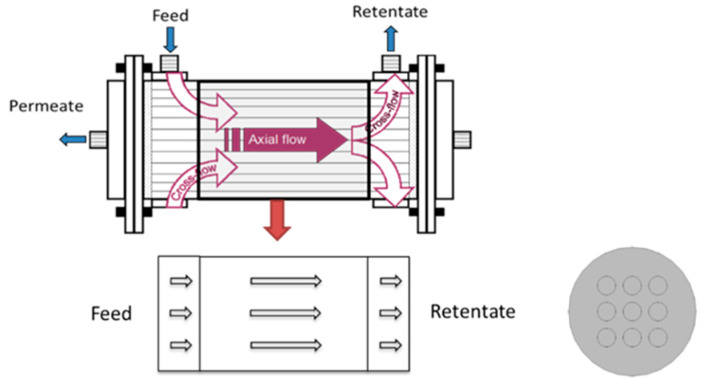
Typical hollow fiber membrane module and flow regions. The domain used for the simulations is indicated by the thick black rectangle in the upper image and the middle rectangle in the lower left image. The lower right image illustrates the module cross-section.

Gas separation membranes are commonly produced in a hollow fiber form. Gas separation modules are formed by creating fiber bundles, embedding bundle ends in a tubesheet to permit separating fluid introduction and removal from the fiber lumen and shell space, and placing the bundle in an enclosing case (see Figure 1). Module performance is influenced by material and operational design variables including gas permeance, selectivity, fiber size dimensions, and operating pressures. Ideal performance models can provide good estimates of performance, but several factors can introduce inefficiencies that dramatically reduce performance. Attempts to account for module inefficiency include introducing the effects of: membrane material property variations (permeance, selectivity, and size); fluid distribution from inlet manifolds into the bundle and collection from outlet manifolds; and fluid distribution within the module and its relationship to membrane packing and module aspect ratio. These issues have been examined for hollow fiber modules to varying degrees [5].

A significant body of work demonstrates that in hollow fiber modules, fiber size variation can have a detrimental impact on performance and staging can partially mitigate the effect [6,7,8]. The literature also partially addresses fluid distribution in the lumen [9] and in the shell-side [10,11,12,13,14,15,16,17]. This work focuses primarily on shell-side flows and the impact of random fiber packing on shell-side mass transfer coefficients for liquid separations.

The effects of design features for introducing and removing fluid from the shell have also been examined [18,19,20]. The most common design uses a distribution collar around the fiber bundle, which communicates through a single port to an external supply or product line as illustrated in Figure 1. The collar may possess diverters or a series of openings to help promote uniform flow into or out of the bundle. However, theoretical and experimental work to characterize the effectiveness of these designs is limited.

Theoretical predictions indicate fluid distribution is uniform if fiber packing is uniform up to the external case [19,20]. However, experimental measurements [18] and theoretical simulations [20] suggest fluid bypassing between the periphery of the fiber bundle and the enclosing case can be detrimental. We are not aware of any work that addresses the impact of fiber packing adjacent to the case on module performance.

This work quantifies the effect of nonuniform packing at the fiber bundle–case interface on gas separation module performance. High-fidelity simulations of fluid flow and mass transfer within a hollow fiber membrane module are developed that capture the potential detrimental effects of non-ideal fluid distribution on module performance. The results can be used to inform carbon capture module manufacturing specifications and guidelines.

Specifically, the effect of packing between the fiber bundle and case is evaluated using full three-dimensional models to simulate module gas separation performance for a shell-fed module. The analysis focuses on the axial flow region between the crossflow regions near the distribution collars by assuming: (1) gas permeation in the crossflow regions is small relative to that in the axial flow region and (2) the axial flow is well developed before entering the axial flow region, as illustrated in Figure 1. Additionally, an equivalent planar bundle (EPB) method is developed based on a simplified three dimensional geometry for a planar bundle derived from the full circular bundle. The EPB is validated against full 3-D circular bundle simulations. Use of the EPB can dramatically reduce computational costs compared to full three-dimensional simulations.

## 2. Simulation Methodology

### 2.1. Theoretical Background

#### 2.1.1. Governing Equations

Module performance is evaluated by solving the governing equations for conservation of momentum and mass for specific module geometries over a range of feed flow rates. The conservation of momentum and continuity equations for steady flows are given by Equations (1) and (2), respectively [21]:

(1) $$\rho \left(\vec{u}\cdot \nabla \right)\vec{u}=\nabla \cdot \left[-p\overset{↔}{I}+\overset{↔}{K}\right]+\vec{F}$$

(2) $$\nabla \cdot \left(\rho \;\vec{u}\right)=0$$

where ρ is the density, $\vec{u}$ is the velocity vector, p is the absolute pressure, $\vec{F}$ is the volume force vector, $\overset{↔}{I}$ is the unit tensor $\delta_{ij}$ and $\overset{↔}{K}$ is the stress tensor.

The conservation of mass equation is given by Equation (3):

(3) $$\nabla \cdot \vec{J_{i}}+\nabla \cdot \left(\rho \omega_{i}\vec{u}\right)=0$$

where $\vec{J_{i}}$ is the relative mass flux vector of component i, and $\omega_{i}$ is the mass fraction.

#### 2.1.2. Constitutive Laws

The stress tensor in Equation (1) in the case of a fluid with internal friction only due to shear stress is given by:

(4) $$\overset{↔}{K}=\mu \left(\nabla \vec{u}+{\left(\nabla \vec{u}\right)}^{T}\right)-\frac{2}{3}\mu \left(\nabla \cdot \vec{u}\right)\overset{↔}{I}$$

where μ is the dynamic viscosity.

Equations (5)–(8) are used to calculate the mass flux $\vec{J_{i}}$ based on the Stefan–Maxwell diffusion model:

(5) $$\vec{J_{i}}=-\rho \omega_{i}\overset{n_{c}}{\underset{k=1}{\sum }}D_{ik}\vec{d_{k}}$$

(6) $$\vec{d_{k}}=\nabla x_{k}+\frac{1}{p}\left[\left(x_{k}-\omega_{k}\right)\nabla p\right]$$

(7) $$x_{k}=\frac{\omega_{k}}{M_{k}}M_{n}$$

(8) $$\frac{1}{M_{n}}=\overset{n_{c}}{\underset{k=1}{\sum }}\frac{\omega_{k}}{M_{k}}$$

where $n_{c}$ is the number of components, $D_{ik}$ are the binary diffusivities for species i and k in the multicomponent mixture, $\vec{d_{k}}$ is the diffusional driving force, $x_{k}$ is the mole fraction, $M_{k}$ is the molar mass, and $M_{n}$ is the average molar mass.

### 2.2. Numerical Setup

#### 2.2.1. Domain Setup and Boundary Conditions

**Figure 2:**
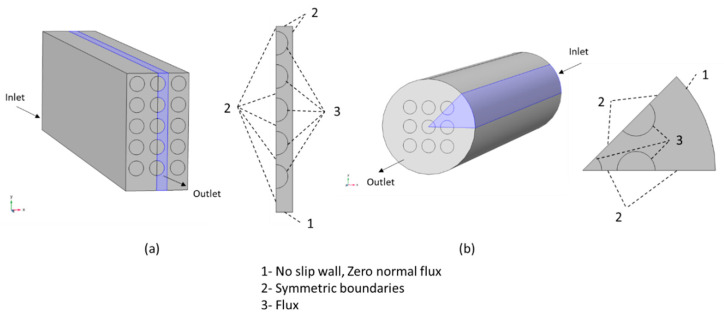
Boundary conditions used in the model: (**a**) planar bundle; (**b**) circular bundle.

Figure 2 illustrates the domain and boundary conditions used for simulations of fiber bundles. At the inlet, the velocity and gas composition are specified as the laminar, well-developed axial velocity, $u_{\;z0}\left(x,y\right)$, and a fixed, uniform feed mole fraction, $x_{Fi}$, respectively:

(9) $$u_{z}\left(z=0\right)=u_{\;z0}\left(x,y\right);\;u_{x}\left(z=0\right)=u_{y}\left(z=0\right)=0$$

(10) $$x_{i}\left(z=0\right)=x_{Fi}$$

where $u_{z}$ is the retentate axial velocity and $x_{i}$ is the mole fraction of component i in the retentate. No-slip, zero normal flux boundary conditions are applied along the case (boundary 1), and symmetry boundary conditions are applied to give lines of symmetry for the planar and circular bundles (boundary 2). For the planar bundle, symmetry reduces the solution domain to a single column of fibers as shown in Figure 2a. For the circular bundle, symmetry reduces the solution domain to one-eighth of the bundle as shown in Figure 2b. For planar bundles, two fiber configurations are considered: square and equilateral triangular.

Assuming a solution–diffusion transport mechanism [22], the membrane is treated as a boundary condition (boundary 3) in the simulation at which the total gas flux across the membrane is calculated from:

(11) $$q_{T}=\overset{n_{c}}{\underset{i=1}{\sum }}q_{i}=\overset{n_{c}}{\underset{i=1}{\sum }}Q_{i}\left(x_{i}p_{h}-y_{i}p_{l}\right)$$

where $q_{i}$ is the transmembrane molar flux of component i, $Q_{i}$ is the permeance, $y_{i}$ is the permeate mole fraction, $p_{h}$ is the retentate (high) absolute pressure, and $p_{l}$ is the permeate (low) absolute pressure. Along the outer diameter of each fiber, the normal wall velocity $u_{n}=M_{n}q_{T}/\rho$ and the normal total mass flux (convective plus diffusive) of component $i={\left(j_{i}+\rho \omega_{i}u\right)}_{n}=M_{i}q_{i}$. To focus on the effects of flow distribution in the shell only and reduce computational complexity, the lumen pressure is assumed to be sufficiently low to neglect $y_{i}p_{l}$ relative to $x_{i}p_{h}$ in Equation (11).

#### 2.2.2. Numerical Methodology

COMSOL Multiphysics^®^ (CFD and TCS modules) was used to solve the governing continuity, conservation of momentum, and conservation of mass equations for the steady, laminar, isothermal flow of a Newtonian fluid. The length of the fiber is 15 cm and the radius of the fiber is 150 µm. Triangular and quadrilateral finite elements were generated in our model. The local grid densities near the inlet and outlet are higher than other sections. A direct solver was used to solve those equations.

#### 2.2.3. Mesh Independence Studies

**Figure 3:**
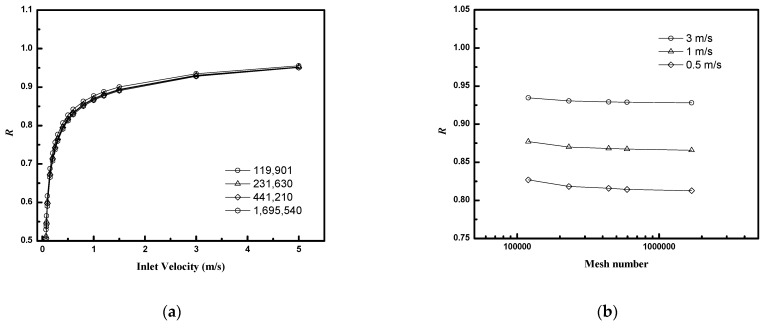
Mesh independence studies (*N* = 16, ϕ=0.4, *D/R_f_* = 4.8, planar bundle): (**a**) ***R*** as a function of inlet velocity for module with different mesh numbers (**b**) ***R*** as a function of mesh numbers for module with different inlet velocity.

COMSOL utilizes the finite element method to discretize the solution domain, using a polygonal mesh, and obtains an approximate numerical solution to the governing partial differential equations. Mesh independence studies are required to ensure that the results are not affected by the chosen spatial discretization of the computational domain. Typically, they consist of calculating quantities of interest in the problem at hand with different mesh resolutions, and identifying a resolution that is computationally feasible and bounds deviations from more refined (but computationally costly) mesh resolutions by an acceptable amount, e.g., 1–5%. Figure 3 illustrates the change in the calculated retentate recovery (***R***) with increasingly finer meshes and associated degrees of freedom. The results change by less than 1% when the degrees of freedom exceed 6,000,000 for each circular model or 500,000 for each planar model.

#### 2.2.4. Darcy’s Permeability

To validate the calculations of fluid flow through axial fiber bundles, the axial Darcy’s permeability in the absence of gas permeation is calculated from:

(12) $$\kappa =\frac{-\mu {\bar{u}}_{z}}{\langle R_{f}{}^{2}\rangle dp/dz}$$

and compared to values reported in the literature [15], where ${\bar{u}}_{z}$ is the superficial axial fluid velocity, $\langle R_{f}{}^{2}\rangle$ is the mean-squared radius of the fibers, and dp/dz is the pressure gradient.

#### 2.2.5. Ideal Counter-Current Module

**Figure 4:**
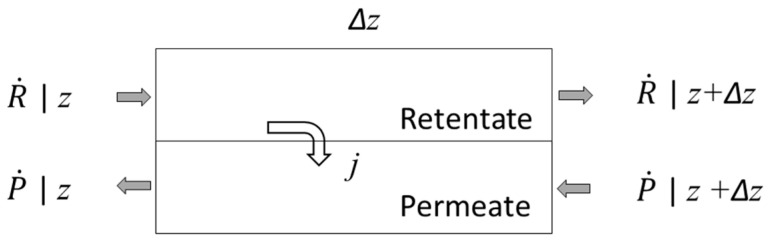
Schematic of ideal counter-current module.

The 3-D module performance simulations are compared on the performance of an ideal counter-current (ICC) module [5]. The ICC model represents the benchmark against which module performance is compared when evaluating the effect of non-idealities as it assumes ideal flow distribution in the lumen and shell and uniform membrane properties. Figure 4 illustrates a mass balance for a differential module length used to develop the ICC performance analysis. ICC performance is calculated from the following mass balance equations:

(13) $$\frac{d{\dot{R}}_{i}}{dz}=-aq_{i}=-aQ_{i}\left(x_{i}p_{h}-y_{i}p_{l}\right)$$

(14) $$\frac{d{\dot{P}}_{i}}{dz}=-aq_{i}$$

With the boundary conditions:

(15) $${\dot{R}}_{i}\left(z=0\right)={\dot{F}}_{i}$$

(16) $$x_{i}\left(z=0\right)=x_{Fi}$$

(17) $$\dot{P_{i}}\left(z=L\right)=0$$

(18) $$y_{i}\left(z=L\right)=\frac{q_{i}\left(z=L\right)}{\sum_{k=1}^{n_{c}}q_{k}\left(z=L\right)}$$

where $R_{i}$ is the molar flow rate of component i in the retentate, ${\dot{F}}_{i}$ is the feed flow rate, $\dot{P_{i}}$ is the permeate flow rate, a is the active membrane area per unit length, and L is the length of the module.

#### 2.2.6. Design Space Explored

**Figure 5:**
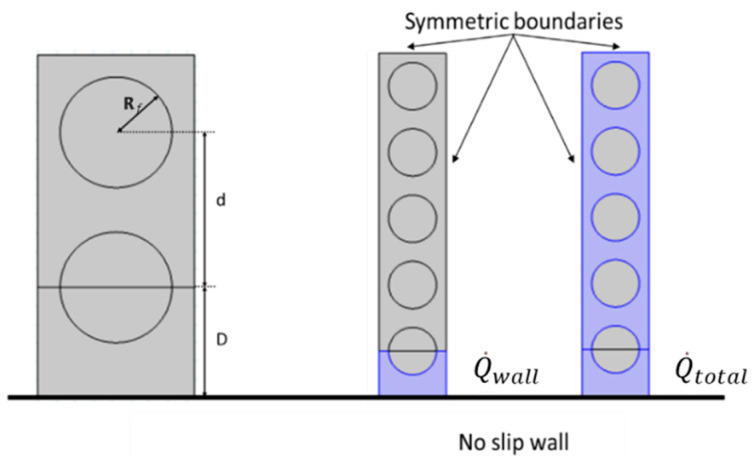
Domain nomenclature for the planar bundle: length definitions (**left**), flow rate definitions ${\dot{Q}}_{wall}$ and ${\dot{Q}}_{total}$ calculated using dark blue regions (**right**), and symmetric boundaries used in the simulations.

**Figure 6:**
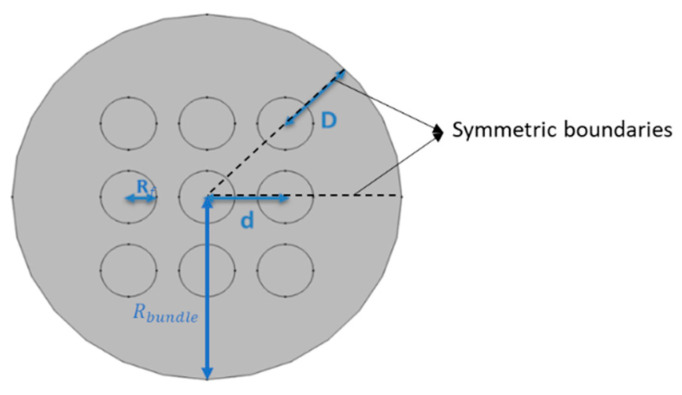
Solution domain nomenclature for the circular bundle and symmetric boundaries.

Table 1 and Figure 5 and Figure 6 indicate the nomenclature used here to describe fiber packing in planar and circular fiber bundles. In this work, the effects of *D/R_f_* and ϕ are studied. The first variable, *D/R_f_*, is a measure of the distance between the case and the outside of the fiber bundle. The second variable, ϕ, is a measure of the distance between fiber centers, *d*. Equation (19) indicates the relationship between the two and reflects the fractional volume occupied by fibers in an infinite bundle:

(19) $$d=\sqrt{\frac{\pi R_{f}{}^{2}}{\phi }}$$

The effects of packing are more readily evaluated for planar bundles due to a reduction in computational requirements. It is anticipated planar bundles will provide a good approximation of circular bundles for sufficiently large bundles where the effects of case curvature can be neglected.

**Figure 7:**
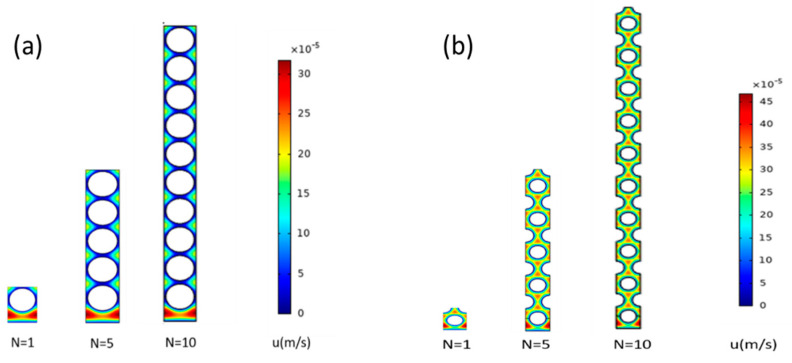
Sample velocity distributions for planar modules in the absence of permeation for: (**a**) square configuration (ϕ=0.6, *D/R_f_* = 2) and (**b**) equilateral triangular configuration (ϕ=0.4, *D/R_f_* = 2). Velocity decreases from red to blue.

Sample two-dimensional axial velocity distributions for planar bundles with regular square and equilateral triangular configurations in the absence of permeation are shown in Figure 7. Fluid bypass at the fiber bundle–wall interface is evident from the higher velocities.

**Figure 8:**
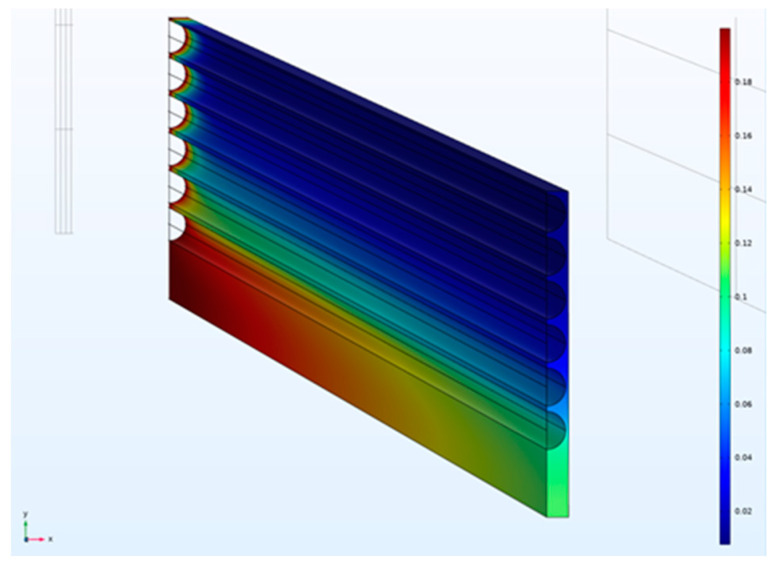
Simulation geometry and sample CO_2_ mole fraction distribution for planar module with square configuration (ϕ=0.6, *D/R_f_* = 4.6). Concentration decreases from red to blue.

**Figure 9:**
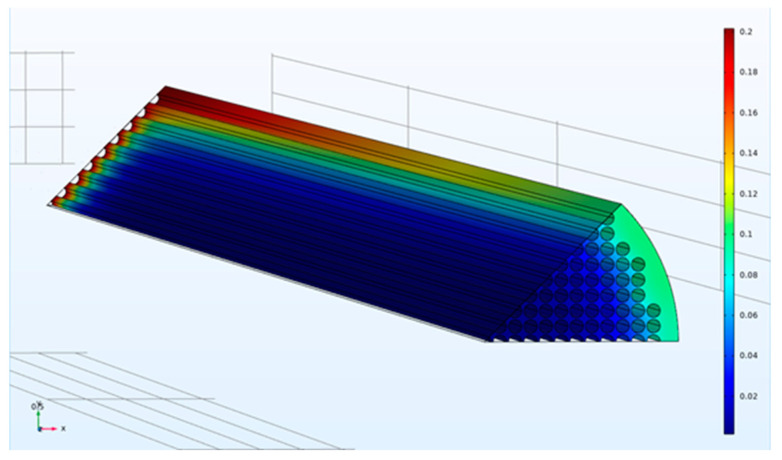
Simulation geometry and sample CO_2_ mole fraction distribution for circular bundle with square configuration (ϕ=0.6, *D/R_f_* = 3). Concentration decreases from red to blue.

Sample three-dimensional CO_2_ mole fraction distributions for a square fiber packing in planar and circular bundles are illustrated in Figure 8 and Figure 9, respectively.

**Figure 10:**
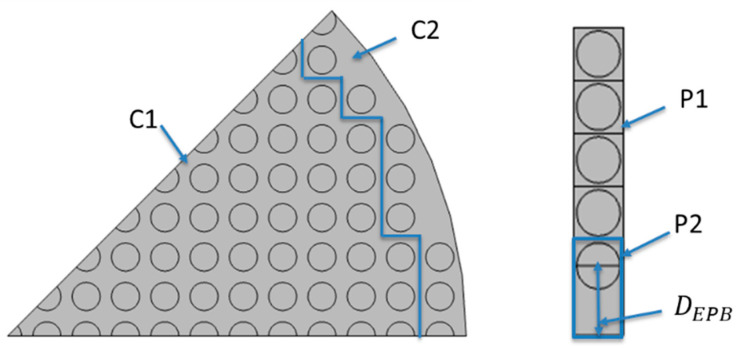
Illustration of the sections used in the EPB method for circular (**left**) and planar (**right**) bundles. Dashed lines divide the circular bundle into horizontal layers and the fiber closest to the wall in each layer is assigned to the wall section.

The EPB approximation is illustrated in Figure 10 where the flow domain is divided into two sections: (1) center and (2) wall. The fiber closest to the wall in each horizontal layer is assigned to the wall section and all other fibers to the center. The value for $D_{EPB}$, the distance from the wall to the closed fiber, in the EPB is calculated from Equation (20) and *N_P1_*, the number of fibers in the region P1, from Equation (21):

(20) $$\frac{A_{F,\;C2}}{A_{C2}}=\frac{A_{F,\;P2}}{A_{P2}}=\frac{\pi *R_{f}{}^{2}}{d*\left(\frac{d}{2}+D_{EPB}\right)}$$

(21) $$\frac{N_{C1}}{N_{C2}}=\frac{N_{P1}}{N_{P2}=1}$$

where $A_{F,\;C2}$ is the total area occupied by fibers in section C2, $A_{C2}$ is the total area of section C2, $A_{F,\;P2}$ is the total area occupied by fibers in section P2, $A_{P2}$ is the total area of section P2, $D_{EPB}$ is the distance from the wall to the center of the closest fiber, and $N_{i}$ is the total numbers of fibers in section i.

## 3. Results and Discussion

The axial Darcy permeability, as a function of packing fraction, was calculated and compared with prior work [15,23]. Simulations were performed for a single unit cell representative of an infinite fiber bundle. The results are presented in Table 2. The agreement between the axial Darcy permeabilities is excellent and helps validate the simulation approach for flow through the bundle.

Table 3 contains membrane properties and module operating conditions used in the simulations. The values for carbon dioxide and nitrogen permeance correspond to values reported in the literature for commercial carbon capture membranes, and are within the region of desired membrane properties for carbon capture [3]. Module performance is characterized by the dependence of retentate recovery *R* (fraction of the feed recovered as the retentate product) and *F* (dimensionless feed flow rate) defined by:

(22a) $$R=\frac{\dot{R}}{\dot{F}}$$

(22b) $$F=\frac{\dot{F}}{Q_{CO2}*a*L*p_{F}}$$

where $Q_{CO2}$ is the CO_2_ permeance, a∗L is the total surface area and $p_{F}$ is the absolute feed pressure. *R* is a measure of the process energy requirements (operating costs) while *F* is a measure of membrane area (capital costs). The values of both are determined as a function of the CO_2_ mole fraction in the retentate product. The effect of the wall gap is quantified by comparing module performance to that for an ideal counter-current model corresponding to an infinite, uniformly packed fiber bundle.

### 3.1. Planar Square Configuration (Velocity Distribution)

*<u>/<u>_∞_* is used to evaluate the effect of poor packing near the case on flow distribution within the bundle. *<u>* is the superficial velocity (flow rate divided by total cross-section area) through a region extending from the fiber wall through a specified number of fibers (*N*) as shown in Figure 5. *<u>_∞_* is the superficial velocity through an infinite fiber bundle without a wall for the same pressure drop. *<u>/<u>_∞_* reflects the increase in flow rate that occurs due to the gap between the fiber bundle and the case. The ratio is highest near the wall where the effect of the wall gap is largest and decreases toward a value of unity as *N* increases. Values are calculated as a function of fiber packing fraction and *D/R_f_* for the square configuration.

**Figure 11:**
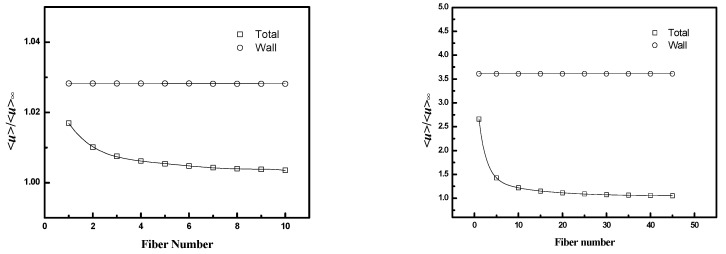

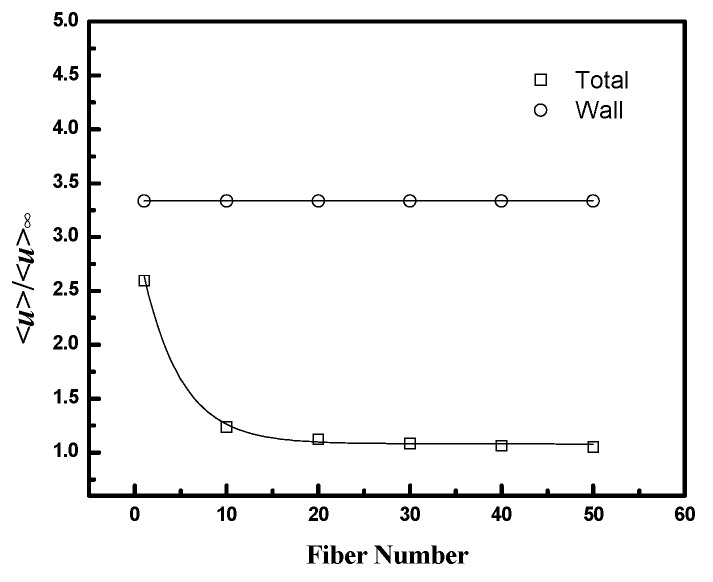
*<u>/<u>_∞_* as a function of fiber number for the whole flow channel (Total) and the channel adjacent to the wall (Wall): (top, **left**) ϕ=0.4, *D/R_f_* = 2, (top, **right**) ϕ=0.6, *D/R_f_* = 2, and (bottom) ϕ=0.4, *D/R_f_* = 3.

**Figure 12:**
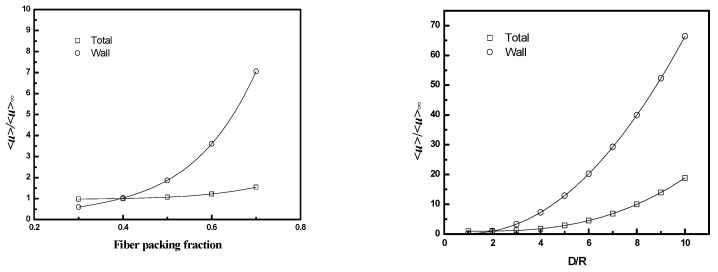
<*u*>/<*u*>_∞_ as a function of fiber packing fraction or *D/R_f_* for square packing; **left** (*D/R_f_* = 2, and fiber number = 10), **right** (ϕ = 0.4, and fiber number = 10).

The results are presented in Figure 11 and Figure 12, along with values for the average velocity in the flow channel adjacent to the wall (the region corresponding to ${\dot{Q}}_{wall}$ in Figure 5). Figure 11 indicates that the velocity ratio for the entire flow channel approaches unity when the number of fibers included in the simulation domain increases, while the ratio in the wall flow channel does not change significantly. The fiber numbers needed for the velocity ratio to be in the range 1 ± 0.05 (i.e., with 5% of the value for an infinite bundle) varied from 10 to 50. The number of fibers increases as the packing fraction increases for a fixed *D/R_f_* or as *D/R_f_* increases for a fixed packing fraction. The velocity ratios for domains containing 10 fibers as a function of packing fraction and *D/R_f_* are illustrated in Figure 12. The results indicate both *<u>_total_/<u>_∞_* and *<u>_wall_/<u>_∞_* increase with either increasing *D/R_f_* for fixed packing fraction or increasing packing fraction for fixed *D/R_f_*.

These results show that the contribution of the bypass flow to the total flow decreases as the number of fibers increases. The magnitude of the bypass flow depends strongly on the distance between the case and the outer fibers of the bundle (*D/R_f_*). The ratio of the bypass flow to the flow through the fiber bundle also depends on fiber packing fraction—for a given *D/R_f_*, as the fiber packing increases the resistance to flow through the interior, the bundle increases, and a greater fraction of the total flow will occur in the bypass region adjacent to the case.

### 3.2. Planar Equilateral Triangular Configuration (Velocity Distribution)

**Figure 13:**
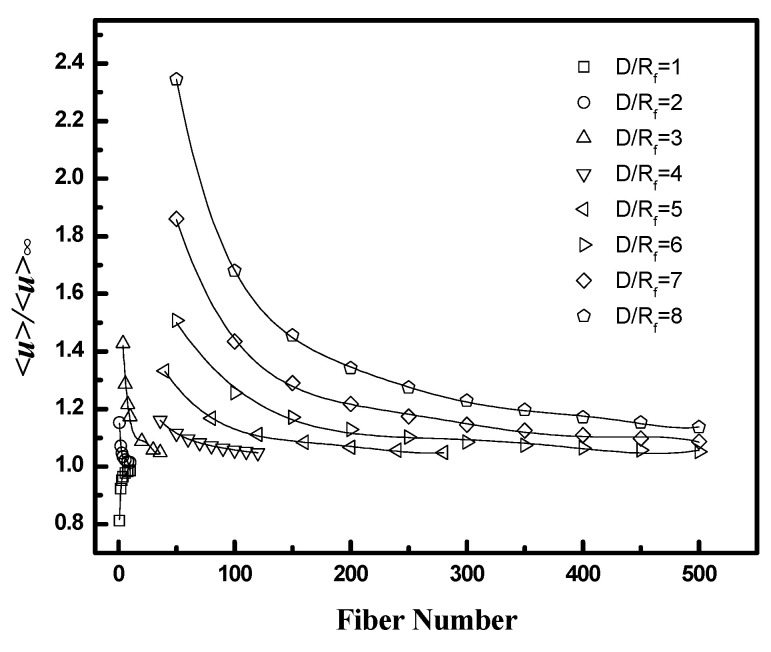
*<u>/<u>_∞_* as a function of fiber number for equilateral triangular configuration with different *D/R_f_*. (ϕ=0.4).

Figure 13 illustrates the effect of fiber number on the results over a broad range of *D/R_f_* values for a packing fraction of 0.4. As for square configuration, the fiber number required for the average velocity ratio to be in the range 1 ± 0.05 increases with *D/R_f_*. The value increases from 10 for *D/R_f_* less than 2 to over 500 for *D/R_f_* greater than 6.

**Figure 14:**
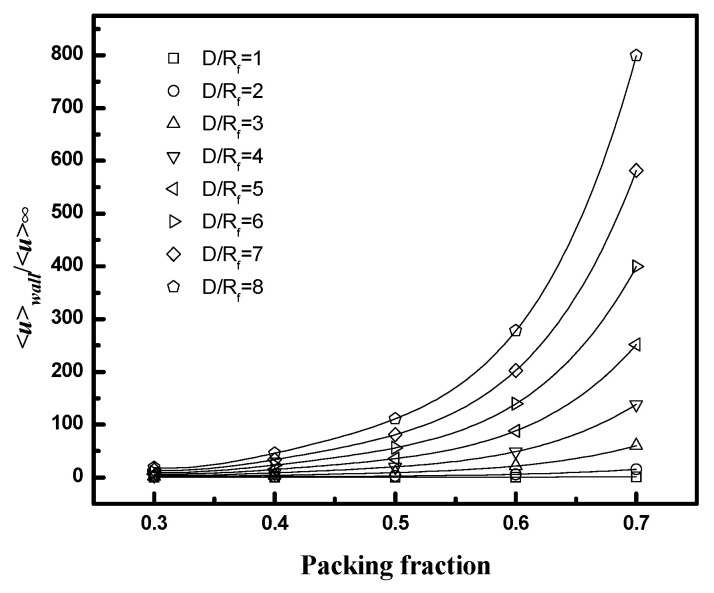
*<u>_wall_/<u>_∞_* as a function of packing fraction and *D/R_f_* for equilateral triangular configuration.

The values for *<u>_wall_/<u>_∞_* as a function of packing fraction (from 0.3 to 0.7) and *D/R_f_* (from 1 to 8) for an equilateral triangular configuration are illustrated in Figure 14. The values depend weakly on fiber number (results not shown) and increase with either increasing *D/R_f_* or packing fraction due to an increasing relative contribution to the total flow from the wall gap region.

### 3.3. Planar Square Configuration

**Figure 15:**
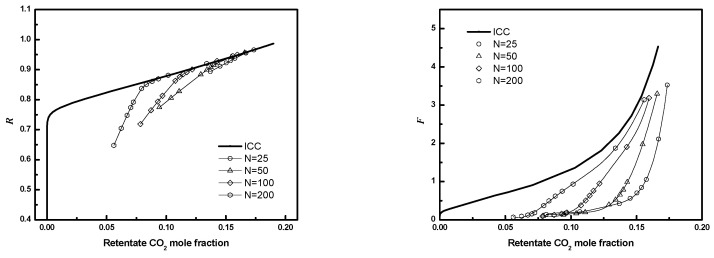
***R*** or ***F*** as a function of CO_2_ mole fraction for module with different fiber numbers (3-D) (*D/R_f_* = 8, ϕ=0.4).

Gas separation module performance as a function of fiber number, packing fraction, and *D/R_f_* for regular packings was evaluated for a module with the membrane properties, operating conditions, and feed specified in Table 3. Figure 15 shows the full 3-D module performance results. For *D/R_f_* = 8 and ϕ=0.4, the calculated module performance is poorer than ICC performance, as the retentate recovery (***R***) is lower for a given retentate product composition—this implies the captured CO_2_ in the permeate will have a lower composition. Additionally, a reduction in recovery implies an increase in operating costs as more pressurized N_2_ is depressurized through permeation and lost to the permeate. This precludes partial recovery of the associated compression energy through an expander as proposed in most carbon capture processes [3].

As expected, the differences become smaller as the fiber number increases from 25 to 200. For sufficiently large bundles, the flow through the bundle will be much larger than the flow through the wall gap, and the effect of the bypass flow will not be significant. Additionally, for sufficiently large bundles, the performance will not depend strongly on the size of the gap created by removing fibers near the wall.

However, for smaller bundles, the impact of poor wall packing is greater as the bypass flow is a greater fraction of the total shell-side flow. The flow through the gap will depend on the fourth power of the equivalent hydraulic diameter of the gap, so small changes in gap size can have a large effect.

The methodology reported in this manuscript provides the quantification of the relative flow through the bundle and the wall gap as a function of fiber number, fiber packing, and gap size. This in turn is used to evaluate module performance and determine how performance is degraded relative to ICC performance for given module properties.

Changes in the dimensionless feed flow rate (***F***) are more significant than the changes in recovery. For a given retentate CO_2_ composition, ***F*** can decrease by a factor of 10 or more. Such a decrease will necessitate an increase in the membrane area and associated capital costs for treating a fixed feed flow.

**Figure 16:**
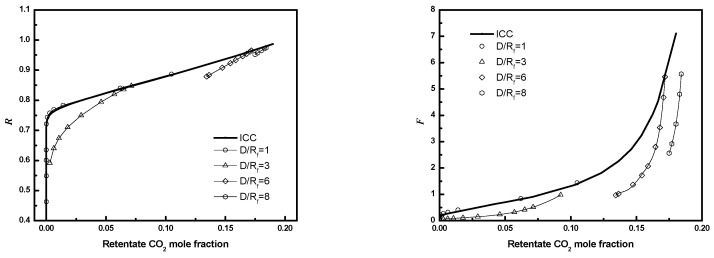
***R*** or ***F*** as a function of CO_2_ mole fraction for module with different *D/R_f_* (3-D, *N* = 25, ϕ=0.6).

The effect of *D/R_f_* on performance for 25 fibers and ϕ=0.6 is illustrated in Figure 16. Performance decreases as *D/R_f_* increases from 1 to 8, suggesting gaps should be minimized.

### 3.4. Circular Square Bundle

**Figure 17:**
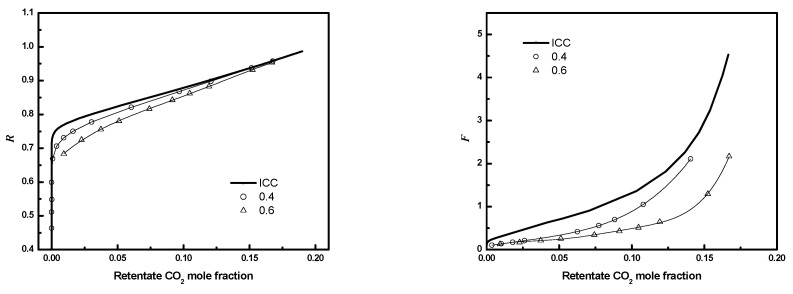
***R*** or ***F*** as a function of CO_2_ mole fraction for module with different packing fraction (3-D) (*N* = 405, *D/R_f_* = 3; circle: ϕ=0.4; up triangle: ϕ=0.6; solid line: ICC).

The performance of full circular fiber bundles is evaluated as a function of fiber number, packing fraction, and *D/R_f_* for regular packings for the conditions in Table 3. Figure 17 illustrates the full 3-D circular square configuration bundle module performance results. Results are presented as retentate recovery versus CO_2_ composition. The results in Figure 17 correspond to *D/R_f_* = 3, ϕ=0.4 or 0.6, and the number of fibers in the entire bundle is 405. For fixed *D/R_f_*, the module performance improves as the fiber bundle packing fraction becomes smaller and approaches ideal ICC module performance. As observed for planar bundles, the impact of poor packing is greater for compact bundles (larger fiber packing fraction) because the resistance to flow between fibers increases as the inter-fiber distance decreases, while the resistance to flow in the gaps between the bundle and case does not change significantly.

**Figure 18:**
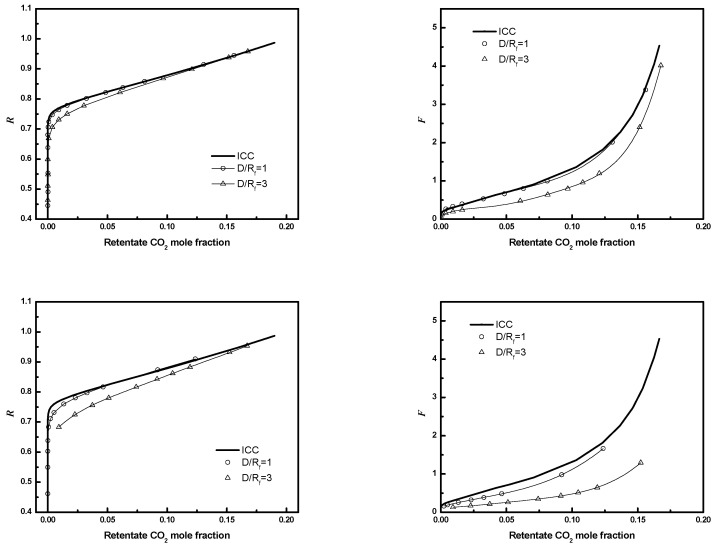
***R*** or ***F*** as a function of CO_2_ mole fraction for module with different *D/R_f_*. Top (*N* = 405, ϕ=0.4), bottom (*N* = 405, ϕ=0.6). Circle, *D/R_f_* = 1; up triangle, *D/R_f_* = 3; solid line, ICC.

The effect of *D/R_f_* on performance is illustrated in Figure 18. The results in Figure 18 correspond to *D/R_f_* = 1 or 3, ϕ=0.4 or 0.6, and number of fibers in the entire bundle equals 405, with the same bundle size and same packing fraction. Module performance improves as *D/R_f_* decreases and the size of the bypass region and its detrimental effect on module performance decrease.

**Figure 19:**
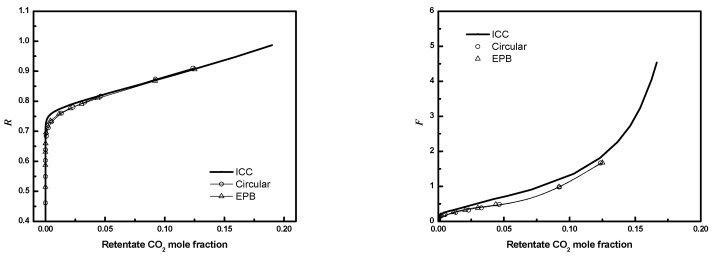
***R*** or ***F*** as a function of CO_2_ mole fraction for full 3-D circular module and EPB module (*N* = 405, *D* = *R_f_*, ϕ=0.6. Circle, circular bundle; up triangle, EPB; solid line, ICC).

**Figure 20:**
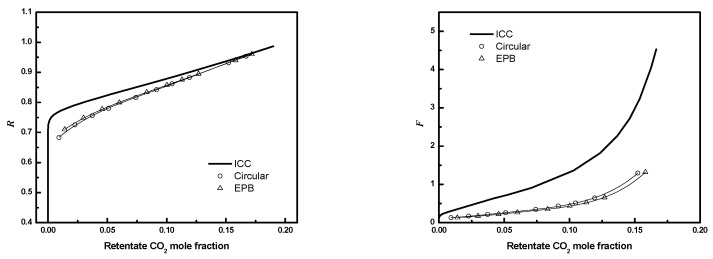
***R*** or ***F*** as a function of CO_2_ mole fraction for full 3-D circular module and EPB module (*N* = 405, *D*/*R_f_* = 3, ϕ=0.6. Circle, circular bundle; up triangle, EPB; solid line, ICC).

**Figure 21:**
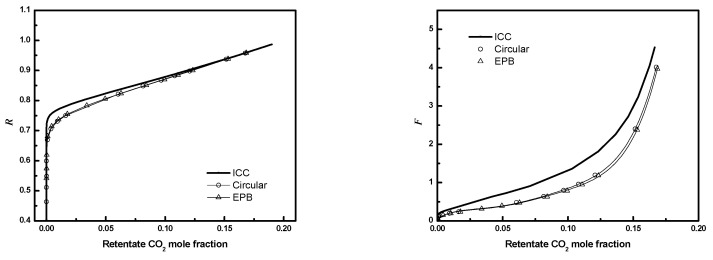
***R*** or ***F*** as a function of CO_2_ mole fraction for full 3-D circular module and EPB module (*N* = 405, *D*/*R_f_* = 3, ϕ=0.4. Circle, circular bundle; up triangle, EPB; solid line, ICC).

To reduce computational costs, an equivalent planar bundle module was introduced previously. Figure 19, Figure 20 and Figure 21 show the module performance comparison between a circular bundle and an equivalent planar bundle for the conditions in Table 3. The results for the circular and equivalent planar bundles agree well despite the differences in the geometry. The result proves that the EPB can be used to simulate performance with reduced computational costs and without sacrificing fidelity.

### 3.5. Circular Triangular Bundle

**Figure 22:**
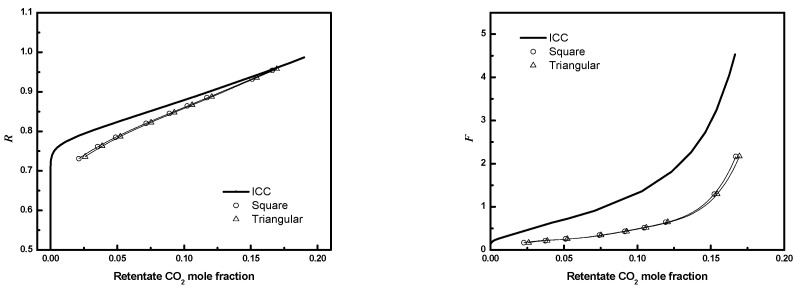
***R*** or ***F*** as a function of CO_2_ mole fraction for full 3-D circular square configuration module and equilateral triangular configuration module (*N* = 405, *D*/*R_f_* = 3, ϕ=0.6. Circle, square configuration; up triangle, triangular configuration; solid line, ICC).

The performances of circular bundles with equilateral triangular configuration are compared to the square configuration for the conditions in Table 3. Figure 22 compares the results for *D/R_f_* = 3, ϕ=0.6, and 405 fibers. The performances of bundles with equilateral triangular configurations are the same as for square configurations for the same conditions. This suggests the packing geometry does not affect module performance significantly for similar operating conditions.

## 4. Conclusions

The performance of shell-fed hollow fiber membrane modules is simulated accounting for poor fiber packing between the fiber bundle and the case. Results are presented for both planar and circular bundles with regular square and equilateral triangular configurations.

Simulations of flow through the bundle clearly indicate the potential for significant bypass between the bundle and the case. The magnitude of the bypass flow depends strongly on how close the bundle is to the case (the distance between the case and the nearest fiber) and the fiber packing fraction. Changes in flow through the bundle arising from the wall gap can extend across thousands of fibers (equivalent to a 35 cm bundle with 1000 fibers and packing fraction of 0.6). The bypass contribution increases as either the fiber packing fraction or distance between the closest fiber and the wall increase. As expected, the contribution of the bypass flow to the total flow decreases as the number of fibers increases.

For both planar and circular bundle modules, significant reductions in module performance can arise relative to an ICC module. These changes are evident in how the module recovery (retentate product flow/feed flow) and dimensionless feed flow change relative to that expected for the ICC. For a fixed retentate product composition, both performance measures are lower than for an ICC. The decrease in recovery implies higher operating costs as more N_2_ is depressurized and lost to the permeate due to permeation, and the associated compression energy cannot be recovered through an expander. The decrease in dimensionless feed flow implies greater capital costs as the membrane area required to process a given feed flow is larger.

The detrimental effects increase as the packing fraction increases, *D/R_f_* increases and module size decreases, as expected due to the relative contribution of the bypass flow to the total flow. Changes in module performance for regular square and equilateral triangular configurations are comparable for the same operating conditions.

An equivalent planar bundle module is also proposed for circular fiber modules. Performance predictions for the EPB are in good agreement with full 3-D circular module predictions. This suggests the EPB can be used to simulate performance with reduced computational costs without sacrificing fidelity.

This work does not address bundle defects except at the wall. One would expect some effect on performance when removing fibers from the middle of the bundle, or other non-uniformities in fiber packing, but the impact of such bundle defects is beyond the scope of the present work.

The methodology reported here can be used to evaluate the impact of poor wall packing on gas separation module performance for other gas separations. While specific results are not provided, similar trends are expected and the impact of poor packing can be quantified.

## Figures and Tables

**Table 1:** Nomenclature for planar and circular fiber bundle domain geometry.

Symbol	Definition
$R_{f}$	fiber radius
$R_{bundle}$	fiber bundle radius
d	distance between fibers
D	distance from wall to center of fiber closest to wall
$\phi =\pi R_{f}{}^{2}/d^{2}$	fiber packing fraction [21]

**Table 2:** Variation of axial Darcy permeability with ϕ for square configuration.

ϕ	κ	Bao [15]	Sangani [23]
0.3	0.234	0.235	0.235
0.4	0.0984	-	-
0.5	0.0445	0.0444	0.0445
0.6	0.0203	-	-
0.7	0.0095	0.0094	0.0094

**Table 3:** Module properties and operation conditions.

Parameter	Value
Feed CO_2_ mole fraction	0.2
Fiber length (*L*)	0.15 m
Fiber outside diameter, OD	3.00 × 10^−4^ m
Selectivity (α)	75
${\mathrm{CO}}_{2}\;\mathrm{permeance},\;Q_{CO2}$	1500 GPU
$N_{2}\;\mathrm{permeance},\;Q_{N2}$	20 GPU
$p_{F}$	2 atm
$p_{l}$	0 atm
$\mathrm{Diffusivity},\;D_{CO2,\;N2}$	1.60 × 10^−5^ m^2^s^−1^
Operating temperature, *T*	298 K

## Data Availability

The data presented in this study are available on request from the corresponding author.

## Disclaimer

This project was funded by the United States Department of Energy, National Energy Technology Laboratory, in part, through a site support contract. Neither the United States Government nor any agency thereof, nor any of their employees, nor the support contractor, nor any of their employees, makes any warranty, express or implied, or assumes any legal liability or responsibility for the accuracy, completeness, or usefulness of any information, apparatus, product, or process disclosed, or represents that its use would not infringe privately owned rights. Reference herein to any specific commercial product, process, or service by trade name, trademark, manufacturer, or otherwise does not necessarily constitute or imply its endorsement, recommendation, or favoring by the United States Government or any agency thereof. The views and opinions of authors expressed herein do not necessarily state or reflect those of the United States Government or any agency thereof.

## References

[1] Friedlingstein P., O'Sullivan M., Jones M.W., Andrew R.M., Hauck J., Olsen A., Peters G.P., Peters W., Pongratz J., Sitch S. (2020). Global Carbon Budget 2020. Earth Syst. Sci. Data.

[2] Allen M. (2018). Framing and Context.

[3] Merkel T.C., Lin H., Wei X., Baker R. (2010). Power plant post-combustion carbon dioxide capture: An opportunity for membranes. J. Membr. Sci..

[4] Wu H., Li Q., Sheng M., Wang Z., Zhao S., Wang J., Mao S., Wang D., Guo B., Ye N. (2021). Membrane technology for CO_2_ capture: From pilot-scale investigation of two-stage plant to actual system design. J. Membr. Sci..

[5] Lipscomb G.G., Sonalkar S. (2004). Sources of non-ideal flow distribution and their effect on the performance of hollow fiber gas separation modules. Sep. Purif. Rev..

[6] Rautenbach R., Struck A., Roks M.F.M. (1998). A variation in fiber properties affects the performance of defect-free hollow fiber membrane modules for air separation. J. Membr. Sci..

[7] Lemanski J., Lipscomb G.G. (2000). Effect of fiber variation on the performance of counter-current hollow fiber gas separation modules. J. Membr. Sci..

[8] Liu B., Lipscomb G.G., Jensvold J. (2001). Effect of fiber variation on staged membrane gas separation module performance. AIChE J..

[9] Park J.K., Chang H.N. (1986). Flow distribution in the fiber lumen side of a hollow fiber module. AIChE J..

[10] Noda I., Brown-West D.G., Gryte C.C. (1979). Effect of flow maldistribution on hollow fiber dialysis—Experimental studies. J. Membr. Sci..

[11] Lemanski J., Lipscomb G.G. (1995). Effect of shell-side flows on hollow-fiber membrane device performance. AIChE J..

[12] Labecki M., Piret J.M., Bowen B.D. (1995). Two-dimensional analysis of fluid flow in hollow-fibre modules. Chem. Eng. Sci..

[13] Bao L., Liu B., Lipscomb G.G. (1999). Entry mass transfer in axial flows through randomly packed fiber bundles. AIChE J..

[14] Lemanski J., Lipscomb G.G. (2001). Effect of shell-side flows on the performance of hollow-fiber gas separation modules. J. Membr. Sci..

[15] Bao L., Lipscomb G.G. (2002). Mass transfer in axial flows through randomly packed fiber bundles with constant wall concentration. J. Membr. Sci..

[16] Wang Y., Chen F., Wang Y., Luo G., Dai Y. (2003). Effect of random packing on shell-side flow and mass transfer in hollow fiber module described by normal distribution function. J. Membr. Sci..

[17] Kim J.C., Kim J.H., Sung J., Kim H.C., Kang E., Lee S.H., Kim J.K., Kim H.C., Min B.G., Ronco C. (2009). Effects of arterial port design on blood flow distribution in hemodialyzers. Blood Purif..

[18] Frank A., Lipscomb G.G., Dennis M. (2000). Visualization of concentration fields in hemodialyzers by computed tomography. J. Membr. Sci..

[19] Ding W., He L., Zhao G., Luo X., Zhou M., Gao D. (2004). Effect of distribution tabs on mass transfer of artificial kidney. AIChE J..

[20] Hao P., Lipscomb G.G., Yampolskii Y., Freeman B. (2010). The effect of sweep uniformity on gas dehydration module performance. Membrane Gas Separation.

[21] Rivero J.R., Panagakos G., Lieber A., Hornbostel K. (2020). Hollow fiber membrane contactors for post-combustion carbon capture: A review of modeling approaches. Membranes.

[22] Baker R.W. (2012). Membrane Technology and Applications.

[23] Sangani A.S., Yao C. (1988). Transport processes in random arrays of cylinders. II. Viscous flow. Phys. Fluids.

